# Inhibition of Vascular Endothelial Growth Factor Receptors 1 and 2 Attenuates Natural Killer Cell and Innate Immune Responses in an Experimental Model for Obliterative Bronchiolitis

**DOI:** 10.1016/j.ajpath.2021.10.018

**Published:** 2021-11-11

**Authors:** Rainer Krebs, Jussi M. Tikkanen, Alireza Raissadati, Maria Hollmén, Kishor Dhaygude, Karl B. Lemström

**Affiliations:** ∗ Translational Immunology Research Program, Transplantation Laboratory, University of Helsinki, Helsinki, Finland; † Department of Cardiothoracic Surgery, Helsinki University Hospital, University of Helsinki, Helsinki, Finland

## Abstract

Obliterative bronchiolitis (OB) after lung transplantation is a nonreversible, life-threatening complication. Herein, the role of vascular endothelial growth factor receptor (Vegfr)-1 and -2 was investigated in the development of obliterative airway disease (OAD), an experimental model for OB. The nonimmunosuppressed recipients underwent transplantation with fully major histocompatibility complex mismatched heterotopic tracheal allografts and received Vegfr1 and -2–specific monoclonal antibodies either alone or in combination, or rat IgG as a control. The treatment with Vegfr1- or -2–blocking antibody significantly decreased intragraft mRNA expression of natural killer cell activation markers early after transplantation. This was followed by reduced infiltration of Cd11b^+^ cells and Cd4^+^ T cells as well as down-regulated mRNA expression of proinflammatory chemokines and profibrotic growth factors. However, blocking of both Vegfr1 and -2 was necessary to reduce luminal occlusion. Furthermore, concomitant inhibition of the calcineurin activation pathway almost totally abolished the development of OAD. This study proposes that blocking of Vegf receptors blunted natural killer cell and innate immune responses early after transplantation and attenuated the development of OAD. The results of this study suggest that further studies on the role of Vegfr1 and -2 blocking in development of obliterative airway lesions might be rewarding.

Chronic lung allograft dysfunction remains a major obstacle to long-term success after lung transplantation. The term chronic lung allograft dysfunction comprises several types of chronic lung dysfunction, including restrictive allograft syndrome and bronchiolitis obliterans syndrome. The latter manifests histologically as obliterative bronchiolitis (OB). OB after lung transplantation is a nonreversible, life-threatening complication, with bronchioles being compressed and narrowed by inflammation and fibrosis due to chronic rejection. The bronchiolar epithelium and the airway wall of the allograft are initially challenged by ischemia-reperfusion injury, and subsequently innate and alloimmune responses are activated. This process may induce a dysregulated repair process and eventually lead to the development of OB, causing gradual occlusion of the airway lumen.^1^

During the development of an immune response, endothelial and epithelial cells play a central role.^2^^,^^3^ Therefore, molecules that activate these cells are promising targets for intervention.^4^ Vascular endothelial growth factor-A (Vegfa) is an angiogenic factor that induces endothelial and epithelial cell activation by binding to Vegf receptor (Vegfr)-1 and -2 on the cell surface^5^^,^^6^ and directly regulates production of chemokines.^7^ Furthermore, VEGFA also has lymphangiogenic and proinflammatory properties and is expressed during rejection events after organ transplantation.^8^, ^9^, ^10^

The main receptors for Vegfa, Vegfr1 and -2, are both expressed by the epithelium and microvascular endothelium of the airway wall as well as by graft-infiltrating mononuclear cells during the development of obliterative airway disease (OAD), a reliable proof-of-concept experimental model for OB.^11^ In addition, *Vegfa* gene transfer enhances the development of OAD, whereas the Vegfr tyrosine kinase inhibitor PTK787 reduces it. Because PTK787 inhibits both Vegfr1 and -2 and other class III protein tyrosine kinases, it was not possible to differentiate between the functions of Vegfr1 or Vegfr2. To gain insight into specific proinflammatory functions of the Vegfrs in the development of OAD, this study analyzes the effect of blocking Vegfr1 and -2 with specific monoclonal antibodies.

## Materials and Methods

### Mice

Specific pathogen-free, inbred, male Balb/c (B/c, H-2^d^) and C57BL/6J (B6, H-2^b^) mice (Harlan, Horst, the Netherlands) that weighed 25 to 30 g and were 2 to 3 months of age were used. Permission for animal experimentation was obtained from the Provincial State Office of Southern Finland. All mice received humane care in compliance with the Guide for the Care and Use of Laboratory Animals.^12^

### Experimental Design

The development of OAD was induced by heterotopically transplanting fully major histocompatibility complex mismatched mouse tracheal allografts from Balb/c donors into C57 recipients. Briefly, donor mice were euthanized with carbon dioxide, the trachea was exposed via thoracotomy, removed above the bifurcation level, and then perfused thoroughly with phosphate-buffered saline that contained 10,000 IU/mL of penicillin and 1 mg/mL of streptomycin (Invitrogen Ltd., Paisley, UK). Recipient operations were performed with the mice under 0.25 mL of anesthesia of using a dilution that contained 0.079 mg/mL of fentanyl citrate, 2.5 mg/mL of fluanisone, and 1.25 mg/mL midazolam (Hypnorm 301960, Janssen-Cilag, Birkered, Denmark; midazolam–Alpharma 400861, Alpharma, Oslo, Norway). The trachea was transplanted into an abdominal skin pouch, and the skin was closed with 5-0 absorbable continuous sutures. For perioperative analgesia, buprenorphine (Temgesic, Schering-Plough, Kenilworth, NJ) was used.

Specific rat anti-mouse monoclonal antibodies^13^ were used to block signaling via Vegfr1 and -2 (kindly provided by ImClone Systems/Eli Lilly and Company). Four experimental groups were set up: allograft recipients were treated with 800 μg^14^ of i) rat IgG (Sigma-Aldrich, St. Louis, MO), ii) rat anti-mouse Vegfr1 neutralizing antibody (MF1, ImClone, New York, NY), iii) rat anti-mouse Vegfr2 neutralizing antibody (DC101, ImClone), or iv) combination of Vegfr1 and Vegfr2 neutralizing antibodies intraperitoneally every third day for 10 doses, starting immediately after the transplantation. The recipients in the 3-day groups received the second dose on day 2 after transplantation and none on day 3. Recipients were euthanized with carbon dioxide and the grafts were removed for histologic, immunohistochemical, and real-time RT-PCR analysis 3 days (*n* = 4 to 5), 10 days (*n* = 5 to 6), and 30 days (*n* = 7 to 8) after transplantation.

In a second set of experiments, the recipients were treated with Vegfr antibodies and simultaneously received a subcutaneous injection of 1 mg/kg per day of tacrolimus (Prograf, FK 506 FR900506, kindly provided by Astellas Pharma, Tokyo, Japan) dissolved in NaCl 0.9%, once per day. These grafts were removed for histologic analysis 30 days after transplantation.

### Histologic Evaluation

Tracheal allografts were excised, embedded in Tissue-Tek (Miles Inc., Elkhart, IN), snap frozen in liquid nitrogen, and stored at −70°C until use. For histologic evaluation, cryostat sections were stained with Mayer's hematoxylin and eosin. Epithelial injury was determined as the percentage of the tracheal lumen not lined by epithelium. Luminal occlusion was evaluated by determining the reduction in luminal area using the ImageJ software version 1.59 (NIH, Bethesda, MD; *http://imagej.nih.gov/ij*).

### Immunohistochemistry

Serial cryostat sections (4 to 6 μm) were stained using the peroxidase ABC method (Vectastain Elite ABC Kit, Vector Laboratories, Burlingame, CA), and the reaction was revealed by 3-amino-9-ethylcarbazole (Vector Laboratories). The sections were counterstained with Mayer's hematoxylin and aquamounted (BDH Ltd., Poole, UK). The following antibodies were used: rat monoclonal to mouse Cd4 (5 μg/mL; catalog number 553043, BD Pharmingen, San Diego, CA), rat monoclonal to mouse Cd8a (5 μg/mL; catalog number 553027, BD Pharmingen), rat monoclonal to mouse Cd11b (5 μg/mL; catalog number 553308, BD Pharmingen), rat monoclonal to mouse Cd31 (5 μg/mL; catalog number ab7388, Abcam, Cambridge, UK), and rabbit polyclonal antibody to mouse lymphatic endothelium-specific hyaluronan receptor 1 (1:1000; a kind gift from Dr. Michael Jeltsch, Drug Research Program, University of Helsinki, Helsinki, Finland). The number of positively staining inflammatory cells, blood vessels, and lymphatic vessels are expressed per allograft cross-section. Specificity controls were performed using the same immunoglobulin concentration of species- and isotype-matched antibodies or irrelevant primary antibodies. Unfortunately, immunohistochemical stainings for NK cells and neutrophils were not of satisfactory quality for analysis. One cross-section per mouse was analyzed for each immunohistochemical staining.

### RNA Isolation and Real-Time Quantitative RT-PCR

Total RNA was isolated from complete tracheal cross-sections using RNeasy kit (Qiagen, Hilden, Germany) and reverse transcribed with a High-RNA-to-cDNA kit (Applied Biosystems, Foster City, CA).

Real-time quantitative RT-PCR reactions were performed on a LightCycler using LightCycler FastStart DNA MasterPLUS SYBR Green I mix (Roche, Basel, Switzerland). The PCR product was measured at the end of each extension period. The number of copies of the gene of interest was calculated from the corresponding standard curve using LightCycler software version 4.0 (Roche) and is given in relation to the correspondent *Actb* molecule numbers. The mRNA expression of the following genes was analyzed with real-time PCR at 3 and 10 days after transplantation: *Angpt2*, *Ccl2*, *Ccl20*, *Ccl21*, *Ccr6*, *Cd52*, *Cd80*, *Cd83*, *Cd86*, *Cxcl1*, *Cxcl3*, *Cxcl9*, *Cxcl10*, *Cxcl11*, *Esm1*, *Ifng*, *Il2ra*, *Il6*, *Il8*, *Il10*, *Il12b*, *Klrb1c*, *Myd88*, *Tgfb1*, and *Xcl1*. In addition, at 3 days after transplantation, the mRNA expression of the following genes was analyzed: *Ccl4*, *Ccr2*, *Cxcl14*, *Cxcr3*, *Egr1*, *Fas*, *Fasl*, *Gzma*, *Has2*, *Hp*, *Il18*, *Klrb1a*, *Klrk1*, *Ncr1*, *Prf1*, and *Sele*. In addition, at 10 days after transplantation, the mRNA expression of the following genes was analyzed: *Ccl3*, *Ccl5*, *Ccl17*, *Ccn2*, *Cd3e*, *Cd4*, *Cd8*, *Cd19*, *Cd20*, *Csf1*, *Csf3*, *Cxcl12*, *Cxcr4*, *Edn1*, *Fgf2*, *sFlt1*, *Hmox1*, *Icam1*, *Ido1*, *Il2*, *Il4*, *Il12a*, *Il16*, *Il17*, *Il21*, *Il22*, *Il23a*, *Il27*, *Mmp9*, *Nos2*, *Pdgfa*, *Pdgfb*, *Selp*, *Vcam1*, and *Xcr1*. For a list of primers used, see Table 1. For an overview of investigated genes and respective results at 3 and 10 days, see Supplemental Table S1.

**Table 1:** Primer Sequences and Gene Bank Accession Numbers

Gene (accession no.)	Forward primer	Reverse primer
*Actb* (NM_007393)	5′-ACCCGCCACCAGTTCGCCAT-3′	5′-GACCCATTCCCACCATCACACCCT-3′
*Angpt2* (NM_007426)	5′-GGTCAACAACTCGCTCCTTCAG-3′	5′-AGGTGGTTTGCTCTTCTTTACGG-3′
*Ccl2* (NM_011333)	5′-CATGCAGGTCCCTGTCATGCTTCTG-3′	5′-CTGGTGAATGAGTAGCAGCAGGTGAGTG-3′
*Ccl3* (NM_011337)	5′-CACTGCCCTTGCTGTTCTTCTCTGTACC-3′	5′-TTCTTGGACCCAGGTCTCTTTGGAGTC-3′
*Ccl4* (NM_013652)	5′-CGTGTCTGCCCTCTCTCTCCTCTTGC-3′	5′-CGCTGGAGCTGCTCAGTTCAACTCC-3′
*Ccl5* (NM_013653)	5′-CAAGGAGTATTTCTACACCAGCAGC-3′	5′-AGCAAGCAATGACAGGGAAGC-3′
*Ccl17* (NM_011332)	5′-CACTTCAGATGCTGCTCCTGGCTG-3′	5′-CCCTGGACAGTCAGAAACACGATGG-3′
*Ccl20* (NM_016960)	5′-GCGTCTGCTCTTCCTTGCTTTG-3′	5′-GCCATCTGTCTTGTGAAACCCAC-3′
*Ccl21* (NM_011124)	5′-GTTTAGGCTGTCCCATCCCG-3′	5′-GGCTGTGTCTGTTCAGTTCTCTTGC-3′
*Ccn2* (NM_010217)	5′-AGCTGGGAGAACTGTGTACGGA-3′	5′-TGCACCATCTTTGGCAGTGC-3′
*Ccr2* (NM_009915)	5′-GCAAAGACCAGAAGAGGGCATTGGATT-3′	5′-GAATACCAGGGAGTAGAGTGGAGGCAGG-3′
*Ccr6* (NM_009835)	5′-CCTGGGTCTTTCGGACTTGGTTCG-3′	5′-GGCGTGGTTCTCTATGTGGATGGG-3′
*Cd3e* (NM_007648)	5′-GGGCTTGCTGATGGTCATTTATTAC-3′	5′-CACAGAAGGCGATGTCTCTCCTATC-3′
*Cd4* (NM_013488)	5′-CGCTTCAGTTTGCTGGTTCTG-3′	5′-TCAGGGTCAGTCTCATCTTGGG-3′
*Cd8* (NM_001081110)	5′-TGCTACCACAGGAGCCGAAAG-3′	5′-GCAGGAAACTTGAAAAAGGAGGG-3′
*Cd19* (NM_009844)	5′-TGGGTTTGGGGGTCTCTTCTGCTTC-3′	5′-CCATCCACCAGTTCTCAACAGCCAG-3′
*Cd20* (NM_007641)	5′-GAGCCTTGGAGCCTTGGAGACCC-3′	5′-CCTTTTGAGGTTCACTTTTGGAGCAGG-3′
*Cd52* (NM_013706)	5′-CAGAGCCCAGGAAGATTTCAGG-3′	5′-TTTGGTGGAGGTGCTGTTTTTG-3′
*Cd80* (NM_009855)	5′-GTCCAAGGCTCATTCTTCTCTTTG-3′	5′-AACGGCAAGGCAGCAATACC-3′
*Cd83* (NM_009856)	5′-ACCGTGGTTCTGAAGGTGACAG-3′	5′-TGGGAAAATGCTTTGTAGTCGTG-3′
*Cd86* (NM_019388)	5′-TGTGTGTGTTCTGGAAACGGAGTC-3′	5′-ACTTAGAGGCTGTGTTGCTGGGC-3′
*Csf1* (NM_007778)	5′-TCCTGTTCTACAAGTGGAAGTGGAG-3′	5′-CATAAAGAGATAGTCCTGTGTGCCC-3′
*Csf3* (NM_009971)	5′-TGGAGCAGTTGTGTGCCACC-3′	5′-TAGAGCAGCCACTCAGGGAAGC-3′
*Cxcl1* (NM_008176)	5′-CCGAAGTCATAGCCACACTCAAGAATGG-3′	5′-TGTTGTCAGAAGCCAGCGTTCACCAG-3′
*Cxcl3* (NM_203320)	5′-CCCAGGCTTCAGATAATCATCAAG-3′	5′-AGGTAAAGACACATCCAGACACCG-3′
*Cxcl9* (NM_008599)	5′-TTTGCCCCAAGCCCCAAT-3′	5′-GTTCTTTTGATGTTTTTTCCCCCTC-3′
*Cxcl10* (NM_021274)	5′-AAATCATCCCTGCGAGCCTATC-3′	5′-GGAGCCCTTTTAGACCTTTTTTGG-3′
*Cxcl11* (NM_019494)	5′-CGAGTAACGGCTGCGACAAAGTTGAAGT-3′	5′-TCCTGGCACAGAGTTCTTATTGGAGGGC-3′
*Cxcl12* (NM_013655)	5′-GCACGGCTGAAGAACAACAACAGACAA-3′	5′-AGGAGCAGAGGAAGTGGCTATGGGC-3′
*Cxcl14* (NM_019568)	5′-AAGCCAAAGTACCCACACTGCGAGG-3′	5′-CCTGGAGTTTTTCTTTCCATGATCGTCC-3′
*Cxcr3* (NM_009910)	5′-CAAAGGCAGAGAAGCAGGCAGCACG-3′	5′-CCAGAAGAAAGGCAAAGTCCGAGGCATC-3′
*Cxcr4* (NM_009911)	5′-TCTGAGGCGTTTGGTGCTCC-3′	5′-GATGATGAAGTAGATGGTGGGCAG-3′
*Edn1* (NM_010104)	5′-TGCCACCTGGACATCATCTGGGTC-3′	5′-CGCACTGACATCTAACTGCCTGGTCTG-3′
*Egr1* (NM_007913)	5′-TAATAGCAGCAGCAGCACCAGC-3′	5′-ATAACTCGTCTCCACCATCGCC-3′
*Esm1* (NM_023612)	5′-CAGCAGCCAAATCTCCCAGCAGG-3′	5′-CGAGCAGCGTTCCCTTCTCCAATC-3′
*Fas* (NM_007987)	5′-TGCTGGAAAAGGAGACAGGATGACC-3′	5′-TCTAAGGTTCTGCGACATTCGGCTT-3′
*Fasl* (NM_010177)	5′-GGAATGGGATTAGGAATGTATCAGC-3′	5′-CAGAGGGATGGACCTTGAGTGG-3′
*Fgf2* (NM_008006)	5′-TCAAGGGAGTGTGTGCCAACCG-3′	5′-ACCAACTGGAGTATTTCCGTGACCG-3′
*sFlt1* (D88690)	5′-TGACTCTCAGACCCCTGGAATCTAC-3′	5′-CTTTTTGCCGCAGTGCTCAC-3′
*Gzma* (NM_010370)	5′-GGTGTTGACTGCTGCCCACTGTAACG-3′	5′-GCCAAATCTCCCCCATCCTGCTACTC-3′
*Has2* (NM_008216)	5′-CCTATGGTTGGAGGTGTTGGAGGAGATG-3′	5′-CCCTGTTGGTAAGGTGCCTGTCGTC-3′
*Hmox1* (NM_010442)	5′-GAATGAACACTCTGGAGATGACACC-3′	5′-TGTGAGGGACTCTGGTCTTTGTG-3′
*Hp* (NM_017370)	5′-AGGAGGAGGAGAAGGAGGAGGAGGTG-3′	5′-TGACCCCAGAGCAGGAGAGTGACAAC-3′
*Icam1* (NM_010493)	5′-TCCGCTGTGCTTTGAGAACTG-3′	5′-TGAGGTCCTTGCCTACTTGCTG-3′
*Ido1* (NM_008324)	5′-AGACTGTGTCCTGGCAAACTGG-3′	5′-GCTGCGATTTCCACCAATAGAG-3′
*Ifng* (NM_008337)	5′-TCAGCAACAGCAAGGCGAAAAA-3′	5′-CTCTTCCCCACCCCGAATCA-3′
*Il2* (NM_008366)	5′-CATGCAGCTCGCATCCTGTGTCAC-3′	5′-TGCTGACTCATCATCGAATTGGCACTC-3′
*Il2ra* (NM_008367)	5′-GAACACCACCGATTTCTGGCTA-3′	5′-GCTTTGTGATTCCCATCCGC-3′
*Il4* (NM_021283)	5′-CCACGGATGCGACAAAAATCACTTGAG-3′	5′-GCGAAGCACCTTGGAAGCCCTACAGAC-3′
*Il6* (DQ788722)	5′-CAAAGCGAGAGTCCTTCAGAGAGAT-3′	5′-GGATGGTCTTGGTCCTTAGCCA-3′
*Il8* (NM_011339)	5′-CCATGCTCCTGCTGGCTGTCCTTAAC-3′	5′-CGGTGGAAATTCCTTTTGTTTGAGGTCC-3′
*Il10* (NM_010548)	5′-CAAGGCAGTGGAGCAGGTGAAGAGTG-3′	5′-CGAGGTTTTCCAAGGAGTTGTTTCCG-3′
*Il12a* (NM_001159424)	5′-CCCTGTGCCTTGGTAGCATCTATGAGG-3′	5′-CACCCTGTTGATGGTCACGACGC-3′
*Il12b* (NM_001303244)	5′-GCTTCTTCATCAGGGACATCATCAA-3′	5′-GAGGAACGCACCTTTCTGGTTACA-3′
*Il16* (NM_010551)	5′-CCATAAACAGGATTTTCAAAGGGAC-3′	5′-GATGACATTCCAGGCTTCAAACC-3′
*Il17a* (NM_010552)	5′-CCCTCAGACTACCTCAACCGTTCCACG-3′	5′-CCCACACCCACCAGCATCTTCTCG-3′
*Il18* (NM_008360)	5′-TGGCTGTGACCCTCTCTGTGAAGGATAG-3′	5′-CTCCATCTTGTTGTGTCCTGGAACACG-3′
*Il21* (NM_021782)	5′-CATTCATCATTGACCTCGTGGC-3′	5′-CTTTGGGTGTCCTTTTCTCATACG-3′
*Il22* (NM_016971)	5′-AATGCTTGCGTCTGAGCGAG-3′	5′-TCTTTTTCAACTGAGCCAGGTTTC-3′
*Il23a* (NM_031252)	5′-GGACTCAGCCAACTCCTCCAGCCAGAG-3′	5′-CGAAGGATCTTGGAACGGAGAAGGGG-3′
*Il27* (NM_145636)	5′-TGCTTCCTCGCTACCACACTTC-3′	5′-CCTCCTCCTCCTTTGAACATTTG-3′
*Klrb1a* (NM_010737)	5′-TCCAACACTTGGGAGGAAGGTCTAGTTG-3′	5′-TCCTTTTGGCAGATCCAACGGTTG-3′
*Klrb1c* (NM_001159904)	5′-TTCACATTGCCAGACATGAACTGGA-3′	5′-AATCAGGATGGGATTCGCAGTCAG-3′
*Klrk1* (NM_033078)	5′-GCTTGCCATTTTCAAAGAGACG-3′	5′-GACCAGTCCCATCCAGTGATAGG-3′
*Mmp9* (NM_013599)	5′-GCCCTGGAACTCACACGACATC-3′	5′-CGGTTGAAGCAAAGAAGGAGCC-3′
*Myd88* (NM_010851)	5′-CTGACCCCACTCGCAGTTTGTTGG-3′	5′-CTGTAAAGGCTTCTCGGACTCCTGGTTC3′
*Ncr1* (NM_010746)	5′-AGCTCTGGTCAAAGTCGAGCAACCCC-3′	5′-GCTTGTGGCAGTCTTCAGTTGGCAAAAG-3′
*Nos2* (NM_010927)	5′-TGGTCACCTACCGCACCCGAGATG-3′	5′-TGGAGCCGCTGCTGCCAGAAAC-3′
*Pdgfa* (NM_008808)	5′-TAACACCAGCAGCGTCAAGTGC-3′	5′-GCTCATCTCACCTCACATCTGTCTC-3′
*Pdgfb* (NM_011057)	5′-TCTTCCTTCCTCTCTGCTGCTACC-3′	5′-CCCCATCTTCATCTACGGAGTCTC-3′
*Prf1* (NM_011073)	5′-GCGTGTGGGGCTGGATGTGAAC-3′	5′-AAGTTGCGGGGGAGGGCTCTGA-3′
*Sele* (NM_011345)	5′-CGGAACAGCAGTTTTCGGCACAGTG-3′	5′-GCAATGAGGACGATGTCAGGAGTGAGGT-3′
*Selp* (NM_011347)	5′-CTTTTCCTGTGATGAAGGCTCG-3′	5′-ATTCCCAAGAGGCTGAACGC-3′
*Tgfb1* (NM_011577)	5′-TGCTAATGGTGGACCGCAAC-3′	5′-CACTGCTTCCCGAATGTCTGAC-3′
*Vcam1* (NM_011693)	5′-GGATGAACACTCTTACCTGTGCG-3′	5′-CTCCAGATGGTCAAAGGGATACAC-3′
*Xcl1* (NM_008510)	5′-GGTTGTGGAAGGTGTGGGGACTGAAG-3′	5′-CAGGGTTATCGCTGTGCTGGTGGAC-3′
*Xcr1* (NM_011798)	5′-AGCGTACAGACTTGAAACCCTGACATGG-3′	5′-CCAAGACCCACAAAACCAGGCTGTTAC-3′

All gene bank accession numbers are active and available to the public under *https://www.ncbi.nlm.nih.gov/nuccore*.

### Protein-Protein Network Analysis

The STRING database (*http://www.string-db.org*, last accessed March 16, 2021)^15^ was used to construct protein-protein interaction maps with direct (physical) and indirect (functional) interactions between the differentially expressed genes. The protein-protein interaction enrichment *P* value indicates the statistical significance of the network. Proteins with a high degree of interactions were considered to be nodes of central importance.

### Statistical Analysis

All data are expressed as means ± SEM and analyzed by SPSS statistics program version 20 (IBM, Armonk, NY). Two-group comparison was performed using parametric analysis of variance followed by Dunnett's test for data with normal distribution and nonparametric *U*-test for data with nongaussian distribution. *P* < 0.05 was regarded as statistically significant.

## Results

In the experimental OAD model, heterotopic tracheal allografts are transplanted from fully major histocompatibility complex mismatched Balb/c donor to C57 recipient mice. If the recipient mice are left without immunosuppression or other treatment modalities, the tracheal allografts show a progressive infiltration of inflammatory cells in the airway wall peaking at 10 days, and the airway lumen is completely occluded by a myofibroproliferative process by 30 days.^16^

### Blocking of Vegfr2 Significantly Increases Epithelial Injury in Tracheal Allografts

**Figure 1:**
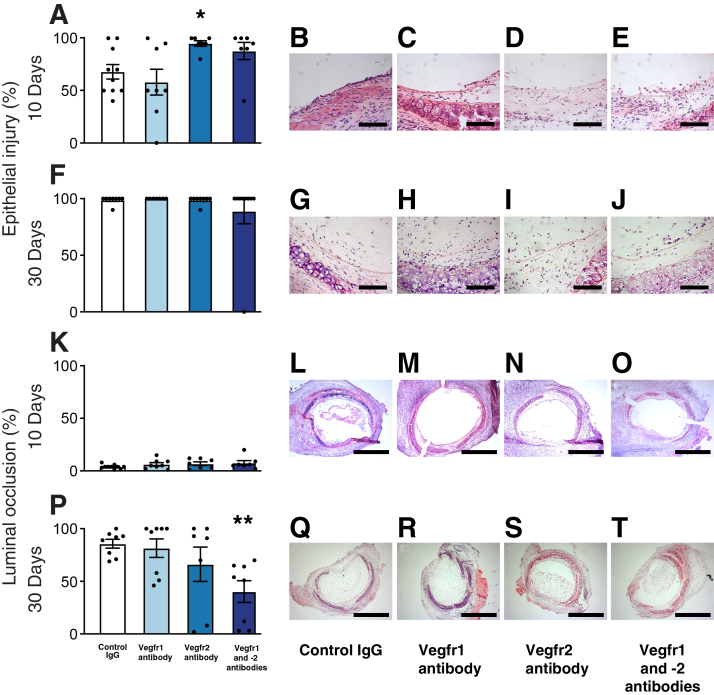
The effects of vascular endothelial growth factor receptor (Vegfr) inhibition on epithelial injury (**A** and **F**) and on the development of luminal occlusion (**K** and **P**) in mouse tracheal allografts. The fully major histocompatibility complex mismatched recipients of tracheal allografts were left without immunosuppression and received rat IgG or neutralizing monoclonal antibodies against Vegfr1, Vegfr2, or both for 10 or 30 days. **A:** Blocking Vegfr2 increases epithelial injury 10 days after transplantation. **P:** Blocking of both Vegfr1 and Vegfr2 significantly reduces the development of luminal occlusion 30 days after transplantation. **B**–**E**, **G**–**J**, **L**–**O**, and **Q**–**T**: Histologic cryostat sections were counterstained with hematoxylin and eosin. Data are expressed as means ± SEM. *n* = 7 to 10 per group. ∗*P* < 0.05, ∗∗*P* < 0.001 versus controls (*U*-test). Scale bars: 100 μm (**B**–**E** and **G**–**J**); 1000 μm (**L**–**O** and **Q**–**T**).

The effect of inhibition of the Vegfr1 and -2 activation pathway with neutralizing antibodies on the epithelial injury of tracheal allografts in nonimmunosuppressed recipients was investigated. While blocking of Vegfr1 signaling had no effect on epithelial injury, inhibition of Vegfr2 signaling significantly increased the epithelial injury of tracheal allografts at 10 days (*P* < 0.05) (Figure 1, A–E). Although the effect of blocking of both Vegfr1 and Vegfr2 on epithelial injury was similar to blocking of Vegfr2 alone, the effect was not statistically significant because of an outlier (Figure 1A). At 30 days, the epithelium was totally lost from the allografts in all treatment groups (Figure 1, F–J).

### Blocking of Both Vegfr1 and -2 Is Necessary to Reduce Luminal Occlusion in Tracheal Allografts

At 10 days, there was a mild development of luminal occlusion in tracheal allografts in all treatment groups in nonimmunosuppressed recipients (Figure 1, K–O). In recipients treated with rat IgG, the lumen was almost totally occluded 30 days after transplantation (Figure 1, P and Q). Only combined inhibition of both Vegfr1 and -2 significantly decreased the development of OAD in tracheal allografts when compared with rat IgG treated recipients (*P* < 0.0005) (Figure 1, P–T).

### Blocking of Vegfr1 and -2 Does Not Affect Angiogenesis and Lymphangiogenesis in Tracheal Allografts

**Figure 2:**
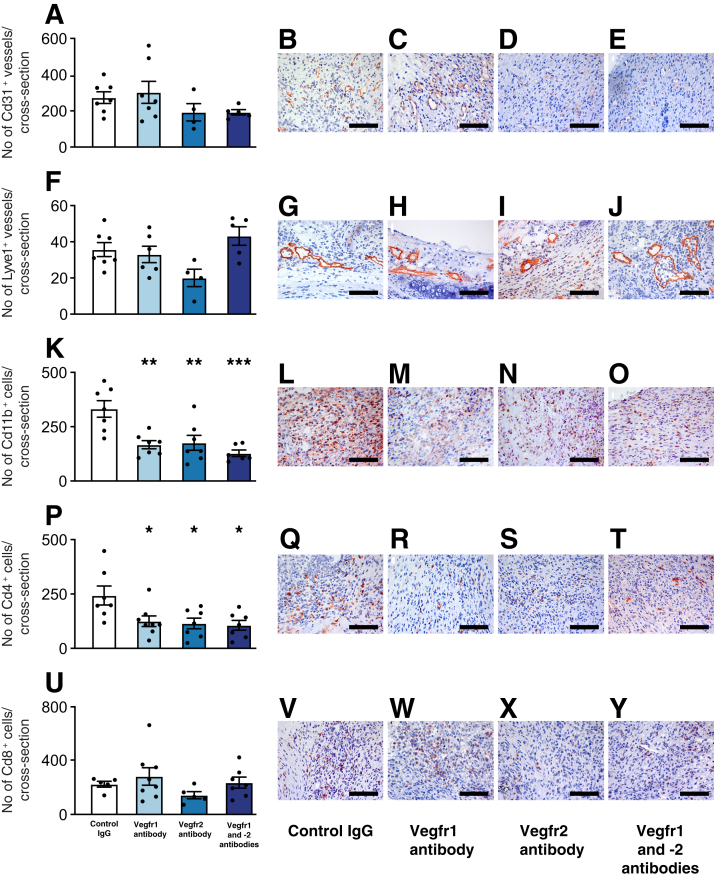
The effect of vascular endothelial growth factor receptor (Vegfr) inhibition on angiogenesis, lymphangiogenesis, and the number of graft-infiltrating inflammatory cells in mouse tracheal allografts 10 days after transplantation. **A** and **F:** Neither the numbers of Cd31^+^ blood vessels (**A**) nor the numbers of lymphatic endothelium-specific hyaluronan receptor 1–positive lymphatic vessels (**F**) are significantly changed by blocking Vegfr1 or Vegfr2. **K**, **P**, and **U**: Blocking Vegfr1 and Vegfr2, alone and in combination, reduces the numbers of graft-infiltrating Cd11b^+^ cells (**K**) and Cd4^+^ T cells (**P**) but has no effect on the number of Cd8^+^ T cells (**U**). **B**–**E**, **G**–**J**, **L**–**O**, **Q**–**T**, and **V**–**Y**: Immunohistochemical cryostat sections were developed with the biotin-avidin system (red) and were counterstained with hematoxylin (blue). Data are expressed as means ± SEM. *n* = 5 to 8 per group. ∗*P* < 0.05, ∗∗*P* < 0.01, and ∗∗∗*P* < 0.001 versus controls (one-way analysis of variance followed by Dunnett's multiple comparison test). Scale bars = 100 μm.

Since this is a nonvascularized model, formation of blood and lymphatic vessel supply to the graft is required after transplantation. All allografts developed a strong peritracheal blood supply by 10 days (Figure 2A). Because Vegfa is known to play a central role in angiogenesis and lymphangiogenesis,^17^ the effect of blocking signaling through Vegfr1 and -2 on the number of Cd31^+^ blood vessel endothelial cells and lymphatic endothelium-specific hyaluronan receptor 1–positive lymphatic vessel endothelial cells in the airway wall of tracheal allografts was investigated. The number of Cd31^+^ blood vessels and lymphatic endothelium-specific hyaluronan receptor 1–positive lymphatic vessels was not significantly affected by blocking of Vegfr1 or -2 or their combination in nonimmunosuppressed recipients 10 days after transplantation (Figure 2, A-E and F-J). These results suggest that the observed effects of Vegfr blocking are not mediated through the inhibition of angiogenesis or lymphangiogenesis.

### Blocking of Vegfr1 or Vegfr2 Reduces the Number of Allograft-Infiltrating Macrophages and Cd4^+^ T Cells

Because Vegfa is also a proinflammatory molecule, blocking of Vegfr signaling may inherently have anti-inflammatory properties.^7^ Therefore, the effects of blocking Vegfr1 and -2 on the infiltration of inflammatory cells into tracheal allografts in nonimmunosuppressed recipients 10 days after transplantation were investigated. Immunohistochemical analysis revealed that blocking Vegfr1 and -2, separately or in combination of both receptors, significantly decreased the number of allograft-infiltrating Cd11b^+^ cells (*P* < 0.005 for single blocking of Vegfr1 and Vegfr2, *P* < 0.0005 for combined blocking of Vegfr1 and -2) (Figure 2, K–O) and Cd4^+^ T cells (*P* < 0.05 for all treatment groups) (Figure 2, P–T). However, none of the treatments had any significant effect on the number of allograft-infiltrating Cd8^+^ T cells when compared with rat IgG-treated recipients (Figure 2, U–Y).

### Blocking of Vegfr1 and Vegfr2 Down-Regulates Intragraft mRNA Expression of Endothelial Cell–Specific Activation Marker and Proinflammatory Chemokines

**Figure 3:**
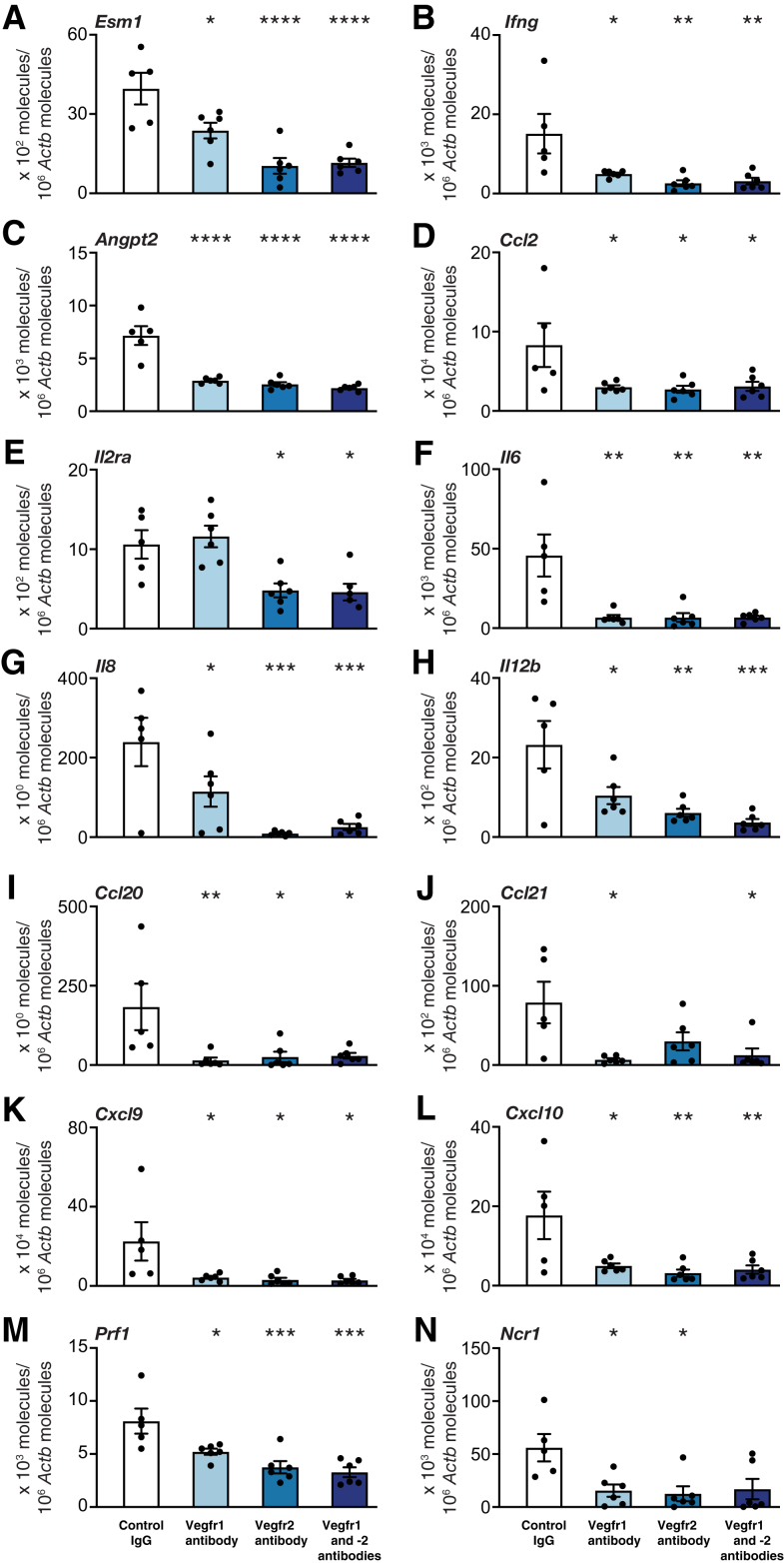
The effect of vascular endothelial growth factor receptor (Vegfr) inhibition on mRNA expression of endothelial cell marker-1 (Esm1) (**A**), proinflammatory cytokines (**B**–**L**), and natural killer (NK) cell–related molecules (**M** and **N**) in mouse tracheal allografts 10 days after transplantation. For proinflammatory cytokines, the quantitative real-time PCR results are shown for proinflammatory molecule interferon γ (Ifng) (**B**); vasculature-destabilizing factor angiopoietin 2 (Angpt2) (**C**); monocyte chemoattractant protein Ccl2 (**D**); T-cell key growth factor receptor Il2 receptor α (Il2ra) (**E**); Th17-related proinflammatory cytokine Il6 (**F**); neutrophil chemoattractant Il8 (**G**); NK cell–activating factor Il12 (**H**); dendritic cell and lymphocyte chemoattractants Ccl20 (**I**) and Ccl21 (**J**); and NK cell and T-cell chemoattractants Cxcl9 (**K**) and Cxcl10 (**L**). For NK cell–related molecules, the quantitative real-time PCR results are shown for NK cell–related cytotoxic effector molecule perforin 1 (Prf1) (**M**) and natural cytotoxicity triggering receptor 1 (Ncr1) (**N**). Data are expressed as means ± SEM. *n* = 5 to 6 per group. ∗*P* < 0.05, ∗∗*P* < 0.01, ∗∗∗*P* < 0.001, and ∗∗∗∗*P* < 0.0001 versus controls (one-way analysis of variance followed by Dunnett's multiple comparison test, except for Il6, Ccl20, and Ccl21, which present data by *U*-test).

The results showed that the numbers of allograft-infiltrating Cd11b^+^ cells and Cd4^+^ effector T cells were significantly reduced by blocking Vegfr1 and -2 alone or in combination at 10 days. Therefore, the effect of the treatment on mRNA expression of the endothelial cell–specific marker 1 (Esm1) as well as innate and adaptive immunity-related factors in tracheal allografts in nonimmunosuppressed recipients was analyzed next (Figure 3). Blocking Vegfr1 and -2, alone and in combination, down-regulated the intragraft mRNA expression of Esm1; Th1 signature cytokine interferon γ (Ifng); vasculature-destabilizing factor angiopoietin-2 (Angpt2); the primary monocyte-recruiting chemokine Ccl2; T-cell activation marker Il2ra (apart from Vegfr1 blocking); proinflammatory cytokine Il6; neutrophil chemoattractant Il8; natural killer (NK) cell–activating factor Il12; neutrophil, dendritic cell, and lymphocyte chemoattractant Ccl20; secondary lymphoid chemokine Ccl21 (apart from Vegfr2 blocking); NK cell and T-cell chemoattractants Cxcl9 and Cxcl10; NK cell–related cytotoxic effector molecule Prf1; and natural cytotoxicity triggering receptor 1 (Ncr1) (apart from combined Vegfr1 and -2 blocking). For an overview of investigated genes and respective results, see Supplemental Table S1.

### Blocking of Vegfr1 and Vegfr2 Down-Regulates Intragraft mRNA Expression of Profibrotic Growth Factors

**Figure 4:**
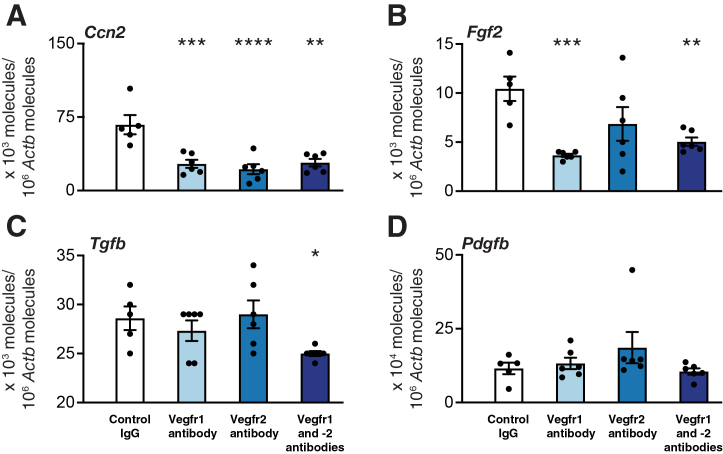
The effect of vascular endothelial growth factor receptor (Vegfr) inhibition on mRNA expression of profibrotic growth factors in mouse tracheal allografts 10 days after transplantation. Quantitative real-time PCR results for connective tissue growth factor (Ccn2) (**A**), basic fibroblast growth factor (Fgf2) (**B**), transforming growth factor β1 (Tgfb1) (**C**), and platelet-derived growth factor B (Pdgfb) (**D**) are indicated. Data are expressed as means ± SEM. *n* = 5 to 6 per group. ∗*P* < 0.05, ∗∗*P* < 0.01, ∗∗∗*P* < 0.001, and ∗∗∗∗*P* < 0.0001 versus controls (one-way analysis of variance followed by Dunnett's multiple comparison test for Fgf2 and Ccn2 and *U*-test for Pdgfb and Tgfb1).

Next, the mRNA expression of profibrotic molecules in tracheal allografts was investigated in nonimmunosuppressed recipients during the peak inflammatory response at 10 days. Blocking of Vegfr1 and -2, alone and in combination, down-regulated the allograft mRNA expression of connective tissue growth factor (Ccn2) (Figure 4A). Blocking Vegfr1 alone and in combination with Vegfr2 down-regulated basic fibroblast growth factor (Fgf2) mRNA expression (Figure 4B), whereas both receptors had to be blocked for significant down-regulation of transforming growth factor β1 (Tgfb1) mRNA levels (Figure 4C). No effect was seen on Pdgfb mRNA levels in any of the treatment groups (Figure 4D). For an overview of investigated genes and respective results, see Supplemental Table S1.

### Blocking of Vegfr1 and Vegfr2 Decreases Intragraft mRNA Expression of NK Cell Markers Early after Transplantation

**Figure 5:**
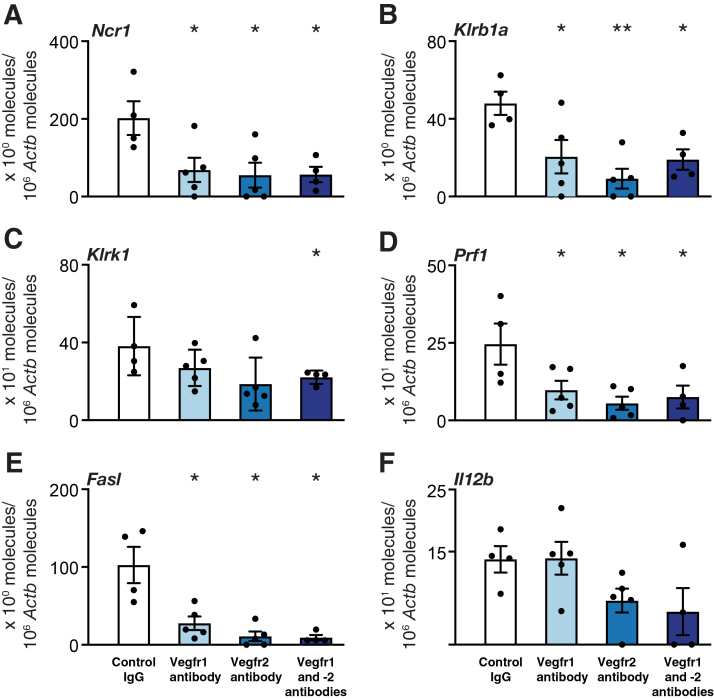
The effect of vascular endothelial growth factor receptor (Vegfr) inhibition on mRNA expression of natural killer (NK) cell activation in mouse tracheal allografts 3 days after transplantation. Quantitative real-time PCR results for NK cell activating receptor 1 (Ncr1) (**A**) and natural killer cell receptor Klrb1a (**B**), for NK cell stress ligand receptor Klrk1 (**C**), for NK cell cytotoxicity effector molecules perforin 1 (Prf1) (**D**) and Fas ligand (Fasl) (**E**), and for NK cell-activating factor Il12 (**F**) are indicated. Data are expressed as means ± SEM. *n* = 4 to 5 per group. ∗*P* < 0.05, ∗∗*P* < 0.01 versus controls (one-way analysis of variance followed by Dunnett's multiple comparison test, except for Fasl, which presents data by the *U*-test).

To get insight into the earlier anti-inflammatory mechanisms of blocking Vegfr signaling, the intragraft mRNA expression of central innate and adaptive immune response–related molecules by quantitative real-time RT-PCR was analyzed at 3 days. In nonimmunosuppressed recipients, blocking of Vegfrs, alone or in combination, down-regulated the mRNA expression of NK cell–activating receptors Ncr1 and natural killer cell receptor Klrb1a, as well as the NK cell–related cytotoxic effector molecules perforin and Fas ligand in all three Vegfr treatment groups when compared with rat IgG-treated recipients (*P* < 0.05; except Klrb1a in Vegfr2 group, *P* < 0.005) (Figure 5). Inhibition of Vegfr1 and -2 signaling was required for down-regulation of NK cell stress ligand receptor Klrk1 mRNA levels (*P* < 0.05) (Figure 5). However, blocking the Vegfrs did not significantly affect the expression of any other investigated innate and adaptive immune-related molecule 3 days after transplantation. For an overview of investigated genes and respective results, see Supplemental Table S1.

### Il6, Ifng, Il8, and Ccl2 Are Key Cytokines in Transmission of Vegfr Blocking Effects

**Figure 6:**
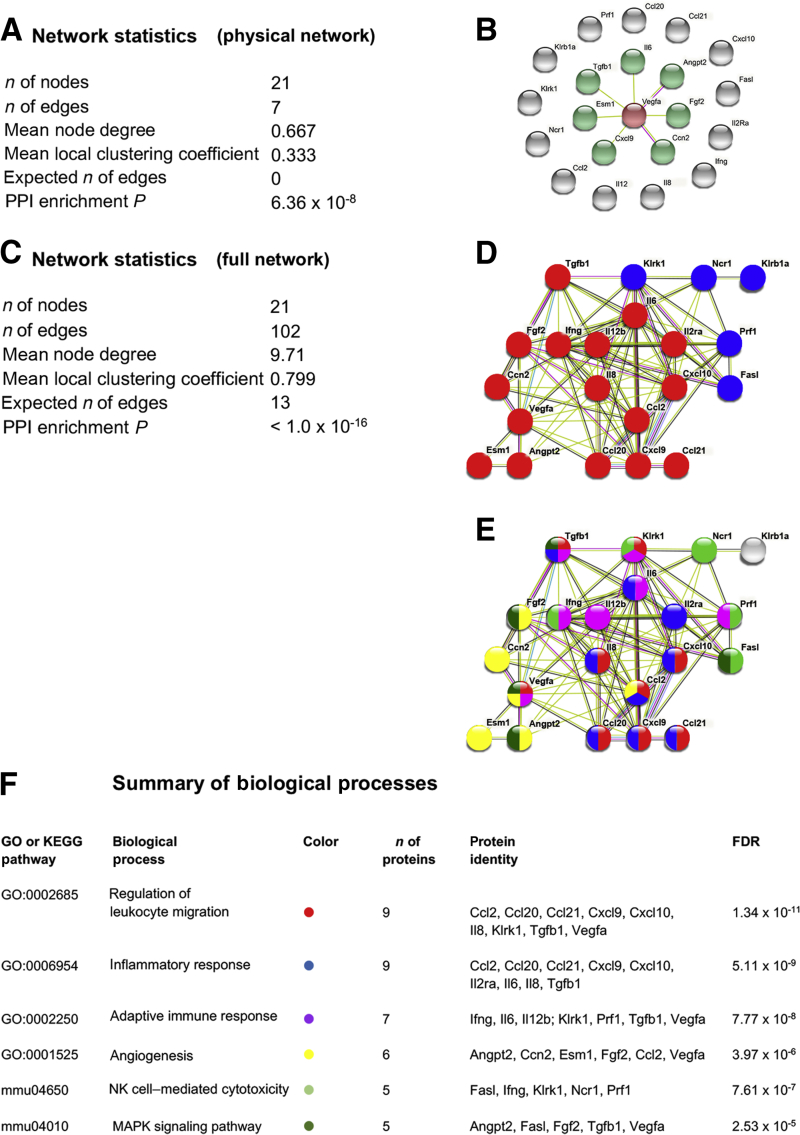
Protein network analysis of genes with differently expressed intragraft mRNA levels after vascular endothelial growth factor receptor (Vegfr) blocking. STRING protein-protein network analysis of differently expressed genes after blocking of Vegfr1 and -2. **A** and **C:** Network statistics for the physical network (**A**) or the full network (**C**) indicate the amounts of nodges and edges, mean node degrees, mean local clustering coefficients, expected numbers of edges, and protein-protein interaction (PPI) enrichment *P* values. **B:** Physical network shows molecules that directly interact with Vegfa. **D:** Blue and red dots in the full network indicate genes with significantly changed expression at 3 and 10 days, respectively. **E:** Colors in the full network indicate the biological processes in which the proteins are involved. Lines between the genes indicate the various types of interaction evidence. **F:** Table of biological processes, indicating the main pathways in which the indicated proteins are involved. Gene ontology or KEGG pathway, number and identity of the proteins, and the false discovery rate are given. Ccn2, connective tissue growth factor; Esm1, endothelial cell–specific marker-1; Fasl, Fas ligand; Fgf2, basic fibroblast growth factor; Ifng, interferon-γ; Klrb, killer cell lectin-like receptor subfamily B; Klrk, killer cell lectin-like receptor subfamily K; MAPK, mitogen-activated protein kinase; Ncr1, NK cell activating receptor-1; Prf1, perforin-1; Tgfb1, transforming growth factor-β1.

For comprehensive networks of direct (physical) and indirect (functional) interactions between the differentially expressed genes affected by Vegfr blocking, the STRING database was used to integrate all known and predicted associations between the genes whose expression was significantly changed in tracheal allografts 3 and 10 days after transplantation. Physical network analysis revealed that Vegf has biochemical bonds directly or through a complex with Esm1, Il6, Angpt2, Cxcl9, Tgfb1, Fgf2, and Ccn2 (Figure 6, A and B). Functional interaction analysis suggested that a strong correlation existed between several measured cytokines with a mean local clustering coefficient of 0.799 (protein-protein interaction enrichment *P* < 1.0 × 10^−16^) (Figure 6, C and D). In addition, based on the higher number of interacting nodes, Il6, Ifng, Il8, and Ccl2 were found to play central roles in this cytokine network. Pathway enrichment analysis was performed to explore the biological significance of the differently expressed genes. The study found that most down-regulated genes belonged to NK cell–mediated cytotoxicity at 3 days and inflammatory response, regulation of leukocyte migration, adaptive immune response, angiogenesis, and the mitogen-activated protein kinase pathway thereafter (Figure 6, E and F).

### Inhibition of T-Cell Activation Pathway Together with Blocking Vegfrs Abolishes OAD Development

**Figure 7:**
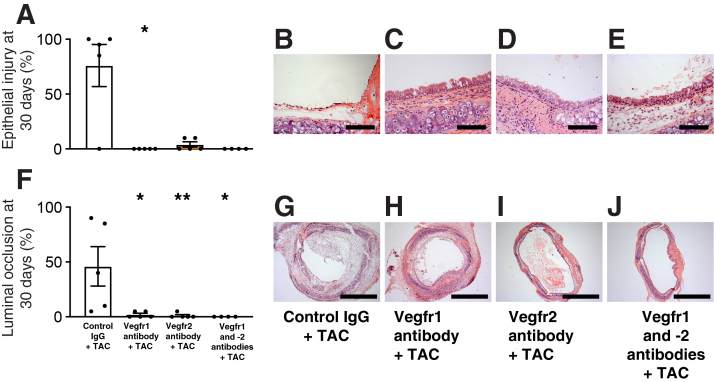
The effect of vascular endothelial growth factor receptor (Vegfr) inhibition in combination with tacrolimus on epithelial injury and on the development of luminal occlusion in mouse tracheal allografts 30 days after transplantation. Recipients received 1 mg/kg per day of tacrolimus (TAC) for 30 days after transplantation. **A:** Blocking Vegfr1 decreases epithelial injury. **F:** Blocking Vegfr1 and Vegfr2, alone and in combination, reduces the development of luminal occlusion. **B**–**E** and **G**–**J**: Histologic cryostat sections were counterstained with hematoxylin and eosin. Data are expressed as means ± SEM. *n* = 4 to 5 per group. ∗*P* < 0.05, ∗∗*P* < 0.01 versus controls (*U*-test). Scale bars: 100 μm (**B**–**E**); 1000 μm (**G**–**J**).

Blocking Vegfr1 and -2 signaling attenuated the intragraft mRNA expression of NK cell and proinflammatory cytokine markers suggested a role in the inhibition of innate immune response. Therefore, the role of blocking the T-cell activation pathway (ie, the adaptive immune response by calcineurin inhibitor tacrolimus in combination with Vegfr inhibition) was investigated next. Tacrolimus has a dose-dependent inhibition on the development of OAD in mice tracheal allografts,^16^ and with the dose of 1 mg/kg per day, tacrolimus treatment reduced epithelial necrosis to 55% and airway occlusion to 60% in tracheal allografts. Inhibition of Vegfrs in combination with 1.0 mg/kg per day of tacrolimus significantly reduced epithelial injury (Figure 7, A–E) only in the Vegfr1 group, whereas it almost totally abolished luminal occlusion (Figure 7) in all three treatment groups at 30 days when compared with rat IgG-treated control recipients.

## Discussion

This study found that blocking of Vegfr1 and -2 signaling in the recipients of tracheal allografts significantly decreased innate and adaptive immune responses and the development of OAD. The results of this study show an association of Vegfr inhibition with reduced levels of endothelial activation markers and an improved state of the epithelium 10 days after transplantation. In addition, Vegfr inhibition elicited down-regulated expression of chemokines for neutrophils, NK cells, macrophages, and T cells. STRING analysis and pathway enrichment showed that the key molecules at 3 days were connected to NK cell activity, whereas at 10 days molecules linked to inflammatory and adaptive immune responses were affected. However, blocking of both receptors was necessary to achieve significant attenuation of airway fibroproliferation. Furthermore, almost complete abrogation of the fibroproliferative development of airway occlusion was observed when Vegfr antibody–treated recipients received a suboptimal dose of tacrolimus. Thus, the results of this study suggest blocking of Vegfr signaling as a novel strategy to limit the chemokine response in the allograft and the development of OB after lung transplantation.

Of the three Vegfr1 ligands, Vegfa and placental growth factor have proangiogenic and proinflammatory properties and increase vascular permeability,^7^^,^^18^, ^19^, ^20^ whereas Vegfb has almost no effect on angiogenesis, vascular leakage, or inflammation.^21^ Vegfa is the main ligand for Vegfr2, with Vegfc binding to it only in its fully processed form.^22^ In contrast to human VEGFD, the murine form does not bind to Vegfr2.^23^ The ligands are generated in response to hypoxia, proinflammatory cytokines, and growth factors by neutrophils, macrophages, T cells, endothelial cells, and many other cell types.^24^, ^25^, ^26^, ^27^, ^28^, ^29^ They drive migration, proliferation, and survival of endothelial cells,^30^ are chemoattractant to monocytes and T cells,^31^^,^^32^ and can enhance T-cell activation and their differentiation into proinflammatory Th1 and Th17 effector cells.^33^ In addition, blockade of VEGFA inhibits transendothelial migration of subsets of mitogen-activated T cells.^34^ In the lung, they are also expressed in epithelial cells,^35^^,^^36^ and VEGFA may regulate the maintenance of vascular structure and function,^37^ as well as play a significant role in the development of OAD.^11^ In general, VEGFA overexpression is a common feature in rejecting allografts.^8^ Although suppressing the signaling pathways of potent angiogenic and lymphangiogenic growth factors,^17^ this study found that the anti-inflammatory effects in the airway wall were observed without any accompanied impact on angiogenesis or lymphangiogenesis. A previous study also found that Vegfa contributes little if at all to the angiogenic activity of tracheal allografts during the development of fibroproliferative changes.^38^

Another important function of Vegfr ligands is the exertion of proinflammatory effects in the allograft.^7^ Macrophages play a central role in the development of OB, as shown in a heterotopic tracheal transplant model.^39^ The reduced influx of macrophages observed in this study may be achieved directly by blocking Vegfr1 signaling on monocytes^40^ or indirectly by reduced signaling through Vegfr2 on endothelial cells and thereby attenuated production of monocyte-attracting Ccl2.^41^ In addition, Cd4^+^ T cells are required for the development of OB,^42^ and the number of allograft-infiltrating Cd4^+^ T cells was also reduced in this study. Functional studies have shown that Vegfa enhances the capture of T cells on activated endothelium and their accumulation in the coronary artery transplant through a direct effect on T cells.^43^ Esm1 or endocan, primarily but not entirely expressed in lung and renal endothelial cells, is a downstream molecule of Vegf. In this study, blocking Vegfr signaling significantly reduced the intragraft mRNA expression of Esm1. Esm1 regulates the leukocyte function–associated antigen 1/intracellular adhesion molecule 1 pathway in endothelial cells and the recruitment of leukocytes to the site of inflammation^44^ and may serve as a marker for inflammation,^45^ endothelial cell activation, and acute renal rejection.^46^ Therefore, it is possible that blocking Vegfr signaling prevented Esm1 activation in endothelial cells of tracheal allografts and thereby reduced the adhesion of leukocytes to endothelial cells and the number of allograft-infiltrating inflammatory cells.

In the tracheal allografts, the inhibition of Vegfr signaling reduced the mRNA expression of proinflammatory molecules Ifng, Angpt2, Cxcl9 and Cxcl10, Ccl2, Il6, Il8, Il12b, Ccl20, and Ccl21 and the profibrotic molecules Ccn2, Fgf2, and Tgfb1. Ifng is produced predominantly by NK cells as part of the innate immune response and by Th1 T cells as part of the adaptive immune response. In macrophages and human bronchial epithelial cells, Ifng strongly induces the expression of Cxcl9 and Cxcl10,^47^^,^^48^ which are efficient chemoattractants for monocytes, NK cells, and T cells.^49^, ^50^, ^51^ Macrophages also produce Ccl2, which recruits monocytes, memory T cells, and dendritic cells to the site of inflammation. Interplay between Il6 and Ifng is central to leukocyte trafficking in inflammation,^52^ and Il6 is also crucial for the development of proinflammatory Th17 immune response implicated in acute and chronic lung allograft rejection.^53^ Il8 is a chemokine for neutrophils and is produced by macrophages, epithelial cells, airway smooth muscle cells, and endothelial cells. Neutrophils have a key role in reperfusion injury and at least in severe forms of acute rejection and OB,^54^^,^^55^ and they are also recruited to the site of hypoxia by Vegfr1.^56^ Of growth factors, Ccn2^57^^,^^58^ and Fgf2 have been implicated in the development of OB.^59^ Ccl20 and the secondary lymphoid tissue chemokine Ccl21 have central roles in regulating the traffic of antigen-presenting cells and T cells^60^, ^61^, ^62^ and thereby direct the connection between innate and adaptive immune responses. These results suggest that blocking Vegf receptor signaling may serve as a checkpoint for innate immune activation and thereby control the development of alloimmune response and OAD.

In transplantation, ischemia reperfusion injury and surgical trauma inevitably occur and induce self-stress molecules to trigger the activation of innate immune response in the first place. This study therefore investigated the mRNA expression of a broad spectrum of inflammation markers and molecules 3 days after transplantation. All molecules that displayed significantly changed mRNA expression levels were related to NK cells: Ncr1 and Klrb1a as activating NK cell receptors, Klrk1 as a receptor for cell stress signals, perforin 1 as a cytolytic NK cell–produced protein, and Fas ligand, which NK cells use to mediate apoptosis. NK cells do not depend on antigen presentation but react to molecules induced by cellular stress and missing self-signals on the allograft tissue. They also induce apoptosis in tubular epithelial cells,^63^ and enhanced levels of Klrk1 mRNA in renal allograft biopsy specimens were associated with allograft rejection.^64^ Furthermore, a recent study shows that NK cells activated through Klrk1 by ligands on endothelial and epithelial cells mediate lung ischemia-reperfusion injury.^65^ Interestingly, this study found that combined blocking of Vegfr1 and -2 significantly down-regulated Klrk1 mRNA levels at 10 days and significantly reduced the luminal occlusion of tracheal allografts 30 days after transplantation.

**Figure 8:**
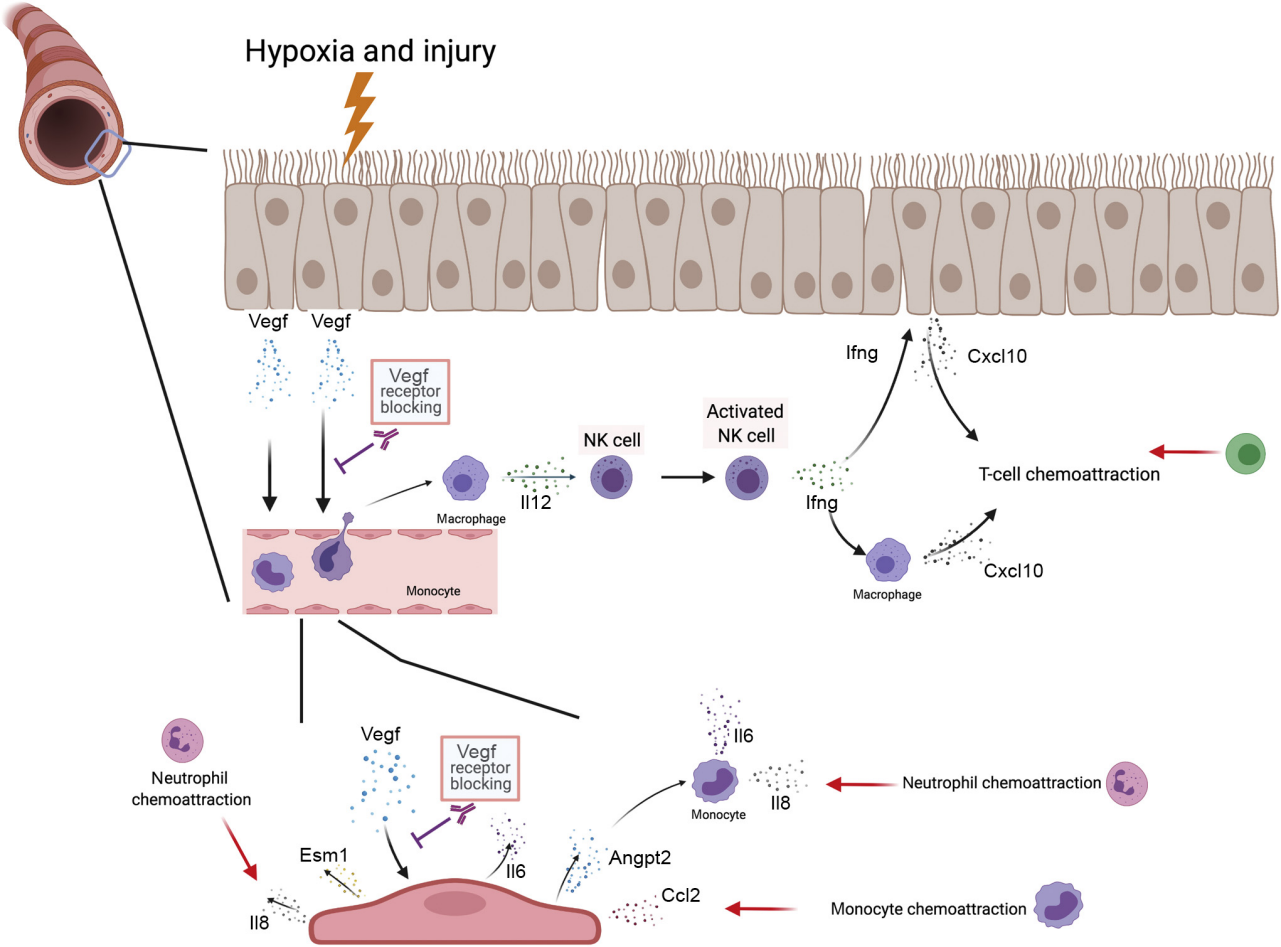
Hypothetical model for immunomodulatory effects of vascular endothelial growth factor A (Vegfa) production after heterotopic trachea transplantation and the correspondent impact of Vegf receptor (Vegfr) signaling blocking. Vegfa can be produced by different sources within the transplanted trachea, such as airway epithelial cells, endothelial cells, and various inflammatory cells. Surgical trauma and hypoxia induce the production and release of Vegfa by epithelial cells. Without intervention, Vegfa acts as a chemoattractant to monocytes, which differentiate into Il12–releasing macrophages on recruitment into the tissue, and can thereby induce the activation of natural killer (NK) cells. The interferon γ (Ifng) generated by these activated NK cells can, in turn, induce epithelial cells and macrophages to release T-cell chemoattractant Cxcl10. Furthermore, Vegfa can activate the endothelial cells of subepithelial blood vessels and trigger their release of Ccl2, monocyte adhesion-promoting endothelial cell–specific marker 1 (Esm1), general emergency warning signal Il6, neutrophil chemoattractant Il8, and angiopoietin 2 (Angpt2), which could induce further production of Il6 and Il8 by monocytes. Blocking of Vegfr signaling prevents the enhanced recruitment of monocytes as well as the activation of blood vessel endothelial cells and their release of proinflammatory cytokines. In other words, blocking of Vegfr signaling may prohibit the development of an extensive adaptive immune response by dampening the early innate immune response (Figure created with biorender.com, Toronto, ON, Canada).

NK cells are a major source of intragraft Ifng, and through Ifng release, subsequently induce allograft cells to produce Cxcl9 and Cxcl10, chemokines for monocytes, T cells, and more NK cells, to launch the adaptive immune response, eventually leading to allograft rejection.^66^ On the other hand, a previous study also suggested a role of Vegfrs in the activation of NK cells.^67^ Furthermore, bronchiolitis obliterans syndrome after lung transplantation has been linked to increased levels of NK cell–derived granzymes, perforin, and Ifng,^68^ and the presence of NK cells in peripheral blood and lung tissue of lung transplant recipients is associated with chronic rejection.^69^ Therefore, it is an intriguing observation that Vegfr inhibition significantly down-regulated the intragraft mRNA expression of receptors and proteins that are important for the activation and action of NK cells early after transplantation. Because activated monocytes provide a stimulus that induces NK cell activation,^70^ a reduced early influx of monocytes due to blocking of Vegfr signaling could be a possible cause of the observed reduced signals of NK cell activity (see hypothetical model in Figure 8). However, although there was a clear trend toward down-regulation of Il12b mRNA when blocking Vegfr2 alone and in combination with Vegfr2 at 3 days after transplantation, the differences failed to meet significance.

Paradoxically, although blocking Vegfr2, alone and in combination with Vegfr1 (in which case significance was prevented by an outlier), resulted in enhanced epithelial loss at 10 days, thereby foreboding enhanced luminal occlusion for the end point, this study still found reduced development of luminal occlusion after combined inhibition of Vegfr1 and -2 at 30 days. However, the same phenomenon was observed by blocking Vegf signaling with a Vegfr tyrosine kinase inhibitor (PTK787) in rats that had received tracheal allografts.^11^ Treatment with PTK787 markedly inhibited epithelial cell proliferation (ie, epithelial regeneration at 10 days) as seen by the reduced number of Ki-67^+^ epithelial cells. Still, receptor inhibition with PTK787 decreased the development of luminal occlusion after 30 days. Considering the known effects of Vegf expression in the setting of transplantation, it is tempting to speculate that the anti-inflammatory effects of Vegfr blocking eventually lead to the reduced development of luminal occlusion. However, considering that blocking the single receptors and blocking both receptors have similar effects on inflammatory cell numbers at 10 days, additional, possibly noninflammatory effects of blocking of both receptors should be investigated to determine the actual causes for the significant decrease of the luminal occlusion at 30 days.

Rodent tracheal transplantation is a technically rather simple and therefore easily reproducible method compared with lung transplantation. Furthermore, baseline immunosuppression is not required for allografts to survive and to develop obliterative changes. However, there are some limitations that must be considered when extrapolating results from this model. First, the trachea is a large airway, and its anatomy differs from bronchioles, which are small, cartilage-free airways and contain club cells that have been shown to be important for keeping the development of OB at bay after lung transplantation. Second, there is no airflow through transplanted tracheas and therefore no contact with the environment. Third, because the tracheal allograft is revascularized by a capillary network developing from the systemic network (ie, in contrast to a transplanted lung), it is not primarily vascularized and therefore experiences strong early ischemic injury. Fourth, the obliterative lesions develop within a month compared with the lesion development in human lung allografts, which takes months or years. However, with the obliterative changes in tracheal allografts reproducibly mimicking the lesions seen in human allograft bronchioles, the mouse heterotopic allograft model is certainly suitable for proof-of-concept studies in the investigation of chronic lung allograft rejection. In addition, this study was unfortunately not able to determine the exact subset of cells that stained positively for Cd11b.

In summary, the results of this study show that modulating activation of the innate immune response early after transplantation could be a crucial step to attenuate the alloimmune response in the development of OAD. Although this study was able to dampen the innate immune response by blocking Vegfr signaling, the development of fibroproliferative occlusion was attenuated, but not totally prevented. However, with the additional administration of a calcineurin inhibitor to abrogate the adaptive immune response, blocking each receptor prevented the loss of epithelium and the development of luminal occlusion almost completely. These findings suggest that further studies on the role of Vegfr inhibition in the development of obliterative airway lesions might be rewarding.
