# Supplementary material for: Inhibition of Vascular Endothelial Growth Factor Receptors 1 and 2 Attenuates Natural Killer Cell and Innate Immune Responses in an Experimental Model for Obliterative Bronchiolitis
Source: Am J Pathol. 2021 Nov 11;192(2):254–69. doi: 10.1016/j.ajpath.2021.10.018 (PMC12178338; doi:10.1016/j.ajpath.2021.10.018)
Supplement: Supplemental Table S1 [file mmc1.docx]

| **timepoint** | 3 days | | | 10 days | | |
| --- | --- | --- | --- | --- | --- | --- |
| **gene** | VEGFR-1 | VEGFR-2 | VEGFR-1+2 | VEGFR-1 | VEGFR-2 | VEGFR-1+2 |
| *Ang-2* |  |  |  |  |  |  |
| *bFGF* | ND | ND | ND |  |  |  |
| *CCL17* | ND | ND | ND |  |  |  |
| *CCL20* |  |  |  |  |  |  |
| *CCL21* |  |  |  |  |  |  |
| *CCL3* | ND | ND | ND |  |  |  |
| *CCL4* |  |  |  | ND | ND | ND |
| *CCR2* |  |  |  | ND | ND | ND |
| *CCR6* |  |  |  |  |  |  |
| *CD3e* | ND | ND | ND |  |  |  |
| *CD4* | ND | ND | ND |  |  |  |
| *CD52* |  |  |  |  |  |  |
| *CD8* | ND | ND | ND |  |  |  |
| *CD80* |  |  |  |  |  |  |
| *CD83* |  |  |  |  |  |  |
| *CD86* |  |  |  |  |  |  |
| *CTGF* | ND | ND | ND |  |  |  |
| *CXCL1* |  |  |  |  |  |  |
| *CXCL10* |  |  |  |  |  |  |
| *CXCL11* |  |  |  |  |  |  |
| *CXCL14* |  |  |  | ND | ND | ND |
| *CXCL3* |  |  |  |  |  |  |
| *CXCL9* |  |  |  |  |  |  |
| *CXCR3* |  |  |  | ND | ND | ND |
| *CXCR4* | ND | ND | ND |  |  |  |
| *E-selectin* |  |  |  | ND | ND | ND |
| *Egr-1* |  |  |  | ND | ND | ND |
| *ESM-1* |  |  |  |  |  |  |
| *ET-1* | ND | ND | ND |  |  |  |
| *Fas* |  |  |  | ND | ND | ND |
| *FasL* |  |  |  | ND | ND | ND |
| *G-CSF* | ND | ND | ND |  |  |  |
| *granzyme A* |  |  |  | ND | ND | ND |
| *haptoglobin* |  |  |  | ND | ND | ND |
| *HAS2* |  |  |  | ND | ND | ND |
| *HO-1* | ND | ND | ND |  |  |  |
| *ICAM-1* | ND | ND | ND |  |  |  |
| *Ido-1* | ND | ND | ND |  |  |  |
| *IFN-γ* |  |  |  |  |  |  |
| *IL-10* |  |  |  |  |  |  |
| *IL-12p35* | ND | ND | ND |  |  |  |
| *IL-12p40* |  |  |  |  |  |  |
| *IL-16* | ND | ND | ND |  |  |  |
| *IL-17A* | ND | ND | ND |  |  |  |
| *IL-18* |  |  |  | ND | ND | ND |
| *IL-2* | ND | ND | ND |  |  |  |
| *IL-21* | ND | ND | ND |  |  |  |
| *IL-22* | ND | ND | ND |  |  |  |
| *IL-23p19* | ND | ND | ND |  |  |  |
| *IL-27p28* | ND | ND | ND |  |  |  |
| *IL-2Rα* |  |  |  |  |  |  |
| *IL-4* | ND | ND | ND |  |  |  |
| *IL-6* | ND | ND | ND |  |  |  |
| *IL-8* | ND | ND | ND |  |  |  |
| *iNOS* | ND | ND | ND |  |  |  |
| *M-CSF* | ND | ND | ND |  |  |  |
| *MCP-1* |  |  |  |  |  |  |
| *MMP9* | ND | ND | ND |  |  |  |
| *Ncr-1* |  |  |  |  |  |  |
| *NKG2D* |  |  |  | ND | ND | ND |
| *NKR-P1A* |  |  |  | ND | ND | ND |
| *NKR-P1C* |  |  |  |  |  |  |
| *P-selectin* | ND | ND | ND |  |  |  |
| *PDGF-A* | ND | ND | ND |  |  |  |
| *PDGF-B* | ND | ND | ND |  |  |  |
| *Prf-1* |  |  |  |  |  |  |
| *RANTES (CCL5)* | ND | ND | ND |  |  |  |
| *SDF-1* | ND | ND | ND |  |  |  |
| *sVEGFR-1* | ND | ND | ND |  |  |  |
| *TGF-β1* |  |  |  |  |  |  |
| *VCAM-1* | ND | ND | ND |  |  |  |
| *XCL1* |  |  |  |  |  |  |
| *XCR1* | ND | ND | ND |  |  |  |

**Supplemental Table S1**

Heatmap of gene expression investigated by real-time PCR. Gray field = non-significant result; green field = significant downregulation of mRNA levels compared to control IgG group; ND = not investigated.
